# President’s Inaugural Address

**Published:** 1907-01

**Authors:** E. Bates Block

**Affiliations:** Atlanta, Ga.; Professor of Nervous and Mental Diseases Atlanta College of Physicians and Surgeons; Visiting Physician to the Presbyterian Hospital, Tabernacle Infirmary, and St. Joseph’s Infirmary


### INAUGURAL ADDRESS DELIVERED BEFORE THE FULTON COUNTY MEDICAL SOCIETY, January 4th, 1906.

By E. BATES BLOCK, M. D.

*Professor of Nervous and Mental Diseases Atlanta College of Physicians and Surgeons; Visiting Physician to the Presbyterian Hospital, Tabernacle Infirmary, and St. Joseph's Infirmary.*

Gentlemen—

According to the custom of this Society, the year begins by an address from the President, whom you have honored by your choice.

There is much of importance for us to accomplish, both in scientific and legislative work, that I thought it best to limit my remarks to those subjects in order to outline our work for the year rather than address you upon some medical subject. This Society, properly conducted, forms for us a post-graduate school of unlimited value. It is of far more value to us than going North for a few weeks each year and having elementary knowledge thrust upon us rapidly without leaving time for contemplation and digestion. Here we may meet the year around and hear papers, which represent the latest knowledge upon each subject.

It is our hope that free use will be made of the books and journals of the Atlanta Medical Library Association, which was founded for this purpose. If each member will read the articles published in these journals during the last few years and give us the resumé of his work he must himself learn much by doing so, and teach us much; but we should not depend entirely upon the published experience of others. Original observation and thought will be excited far more by the study and report of our own cases and observations, and enable us to add our part to the accumulating medical knowledge of the world. In order that our ideas and work may be of benefit beyond our own narrow circle, and to stimulate a desire for accuracy and caution in our statements, we wish this year to make a special effort to have all the papers and the discussions of papers published in a reputable medical journal, which shall become our official organ. The *Atlanta Journal-Record of Medicine* has consented to undertake this task, and to become the official journal of the society.

The greatest aid to our work lies in our possession of a medical library, which enables us to know what the rest of the world is doing. Doctors of other towns are quite as busy as we are and have just as much opportunity for personal experience. With a rapidly growing library of over thirteen hundred bound volumes and an equal number of unbound volumes and an annual subscription to fifty medical journals, representing the best thought and work of the English speaking world, we place ourselves in a position to know more than our neighboring towns and be worthy of the confidence which they have always shown us. While I hold the knowledge of the medical literature of the times as the most important matter for the society, I do not wish to minimize the importance of reporting our personal observations and scientific work, and the demonstration of cases and pathological specimens before the society. Pathological specimens removed at autopsies or in operations should always be shown, when practicable, to the society.

Aside from the medical work of this society, we have another duty to perform: the formation and preservation of medical laws, assuring protection to the public against the imposition of quackery and people who are not registered and legitimate practicians. One of the chief abuses of our times is the requirement of the law that legiitmate practitioners must graduate, stand an examination and pay a license, while another man is allowed to practice medicine without doing any of these, and is even allowed to sue people in our courts and recover fees for his work. Yet the laws of our State are clear upon this point. It is only necessary that some one interested in the subject shall bring the matter before the Grand Jury and be prepared to pay a lawyer to represent the case in order to clear our town of these people. For this purpose, it would be well for us to engage a lawyer to aid our committee, appinted to bring this list of illegitimate practitioners before the Grand Jury and to make an assessment upon the members of this society to defray the expenses. There is, however, a class of illegitimate practitioners here, the Osteopaths, who claim in court that they are not practicing medicine, although they claim this readily enough before the public. It was on this plea that the courts failed to convict them a few years ago. It will be necessary to alter the wording of our law upon this subject, to include all people who by any means, mechanical or chemical, attempt to treat diseases and accept or request remuneration therefor, who should be considered as practicing medicine. An exception must, of course, be made to massage, when nothing more is claimed for it.

One great abuse with which we have to deal is the unethical conduct of many of the pharmacists of Atlanta. Many of the drug stores of our city will sell morphine, cocaine, or any other poisons to people without even asking if they are taking it under a physician's advice. To them it seems to be only a matter of how they can make a few extra cents, without once considering the fate of the poor devil whom they are helping to ruin. Many of our drug stores openly advertise patent medicines under their own names, and yet receive the support of the physicians of Atlanta, who send them patrons.

The evil of counter-prescribing has repeatedly been brought before this society. Here again the druggist seems to think that he has a right to every cent he can get, and for the sake of the money in it will advise a patient what to take and sell it to him without knowing or caring what his disease is, just as the optical companies will sell people glasses when they are suffering from cataract, albuminuric retinitis, or optic atrophy, either with unpardonable ignorance or criminal avarice.

Within ten days I was called to see a young woman who had been prescribed for by a druggist. He gave her Bromo-Seltzer for a headache and told her it was harmless. I was called in on account of cardiac weakness with acute respiratory distress.

In *Collier's Weekly* of December 2nd, 1905, there is an article giving a list of the headache remedies containing acetanilid with a list of twenty-two people who died from poisoning by them. The list included most of the headache remedies we hear about, such as Bromo-Seltzer, Antikamnia, Phenelgin, Cephalgin, etc.

It would be both profitable and interesting to the members of this society to read the articles published from time to time in the last few months in *Collier's Weekly* or the *Ladies' Home Journal* on patent medicine frauds. The morphine in soothing syrups, the cocaine in catarrh cures and the acetanilid in headache powders, should be sufficient to enable us to force the druggists to comply with the laws to label all such medicines as poisons.

The difficulty of obtaining just medical legislation is made painfully clear by *Collier's Weekly* of November 4th, 1905. The patent medicine men pay the newspapers of the United States over forty million dollars a year for their advertisements Their contracts with the papers specify that if anything should appear in such papers adverse to their interests, or if any laws are enacted in their State prejudicial to them, their contract for advertising space at once terminates. Let, then, any one be so bold as to propose or support any measure in a legislature and the Proprietary Association of America writes to the editors of the papers in the State that, should such a law pass, their advertisements cease. The editors begin at once to write articles to prevent these laws, and write also to the members of the legislature that unless they drop the question the newspaper will oppose them for any future election, and if the matter comes to a vote at all the most enthusiastic supporters of the reform measures rarely have the hardihood to vote for the bill which they themselves proposed.

We speak of the trusts as some gigantic monster. Look the facts fairly in the face, and you will see that the newspapers are the greatest menace to the public that exists to-day. They blast characters to fill their columns, wreck healths to fill their pockets, tell the truth when it suits them, lie when it is to their interest, and the public believes them because it is printed. Let a big trust be formed. Newspapers villify it until the trust finds it cheaper to increase the revenues of the paper. Then the newspapers either cease to villify it, or speak kindly of it, and the innocent public never sees the fraud.

The meaning of this is plain. We cannot hope to accomplish anything through the papers unless they are blind to our end. Even when they do not say anything against our proposed measures they can easily say to representatives which way they shall vote. We have but one hope left. That is to organize and cling together and elect our own representatives with the understanding that we shall frame just laws. To do this, we must put aside any personal wishes and vote on public questions as the society decides we shall vote. No political office should be filled in our town, county or State without our society deciding whom we will support and every member voting for him. There are about four hundred physicians in Atlanta, and their votes, with their influence, will at once give us a political power which we can never attain in any other way and will create for us such respect on the part of politicians that they will be ready to lend an ear to our wishes, which they have never done heretofore. This principal will eventually be adopted by other county societies, and the State association, and when this is done, we will be in a position to give instead of begging favors.

The Committee on Legislation has done much during the past year towards getting statistics and data for use this year, and it is earnestly hoped that the members of the medical profession will register their licenses to practice medicine at once, so that this committee may carry into effect their authority to indict unregistered physicians.

It is to be hoped that Dr. Sterling's suggestion, that cases or specimens be demonstrated at every meeting, can be carried out this year, and also his suggestion that the meetings begin promptly at 8 o'clock.

In conclusion I wish to thank you again for the confidence you have shown in me by electing me as your President, and I shall use every effort to show myself worthy of it.

